# Alternative to antibiotic growth promoters: beneficial effects of *Saccharomyces cerevisiae* and/or *Lactobacillus acidophilus* supplementation on the growth performance and sustainability of broilers’ production

**DOI:** 10.3389/fvets.2023.1259426

**Published:** 2023-09-12

**Authors:** Youssef A. Attia, Shereen Basiouni, Nisreen M. Abdulsalam, Fulvia Bovera, Afaf A. Aboshok, Awad A. Shehata, Hafez M. Hafez

**Affiliations:** ^1^Sustainable Agriculture Production Research Group, Agriculture Department, Faculty of Environmental Sciences, King Abdulaziz University, Jeddah, Saudi Arabia; ^2^Institute of Molecular Physiology, Johannes-Gutenberg University, Mainz, Germany; ^3^Clinical Pathology Department, Faculty of Veterinary Medicine, Benha University, Moshtohor, Toukh, Egypt; ^4^Department of Food and Nutrition, Faculty of Human Sciences and Design, King Abdulaziz University, Jeddah, Saudi Arabia; ^5^Sustainable Agriculture Production Research Group, Department of Veterinary Medicine and Animal Production, University of Napoli Federico II, Napoli, Italy; ^6^Department of Poultry Nutrition, Animal Production Research Institute, ARC, Ministry of Agriculture and Land Reclamation, Giza, Egypt; ^7^Structural Biochemistry of Membranes, Bavarian NMR Center, Technical University of Munich (TUM), Garching, Germany; ^8^Institute of Poultry Diseases, Faculty of Veterinary Medicine, Free University of Berlin, Berlin, Germany

**Keywords:** antibiotic growth promoters, blood biochemistry, carcass traits, digestibility, meat quality probiotics, productive performance, symbiotics

## Abstract

Although antibiotics growth promoters (AGPs), including zinc-bacitracin (ZnB), can threaten human health due to developing antimicrobial resistance, as well as drug residue in animal and poultry products, ZnB is still widely used, particularly in developing countries, for the sustainability of poultry farming. The present investigation aims to assess the use of *Saccharomyces cerevisiae* and *Lactobacillus acidophilus,* with or without a prebiotic (mannooligosaccharide, MOS), as alternatives to ZnB. For this reason, 150 one-day-old chicks were grouped into six groups, designated negative control, LA, SC, ZnB, SA + MOS, and LA + MOS (5 replicates of 5 chicks for each group). Chicks kept in the control group were fed the basal diet. Chickens kept in LA and SC groups received *L. acidophilus, S. cerevisiae* at a 1 g/kg diet and 2 g/Kg, respectively. Chickens kept in ZnB received ZnB at 0.5 g/kg. Chicks kept in the SC + MOS and LA + MOS were fed a basal diet containing 2 g *S. cerevisiae* + 1 g MOS/kg or 1 g *L. acidophilus* + 1 g MOS /kg, respectively. The efficacy was assessed based on the growth performance, carcass traits, meat quality, nutrient digestibility, and blood biochemistry composition during the entire trial 1–36 days of age. Results showed that chicks kept in the SC group had greater BW than the control (*p* < 0.05). Chicks kept in the SC, LA, SC + MOS, and LA + MOS consumed less feed than the control and Zn-B groups (*p* < 0.05). Supplementation with *S. cerevisiae* resulted in a better (*p* < 0.05) feed conversion rate (FCR) than the control group. Supplementation with *L. acidophilus* + MOS significantly increased (*p* < 0.05) the relative liver weight compared to those supplemented with ZnB, *S. cerevisiae*, and *L. acidophilus*. In addition, supplementation with ZnB-induced spleen hypertrophy compared to *S. cerevisiae* and *L. acidophilus*-supplemented groups (*p* < 0.05). Plasma, meat, and liver cholesterol, as well as the cholesterol-to-lipid ratio of meat and liver, were significantly decreased (*p* < 0.05) in both SC and LA groups compared to the control group. Our research indicates that adding 2 g/kg of *S. cerevisiae* to broiler feed can effectively replace ZnB and enhance productive performance and economic profits, making it a viable and sustainable option for broiler farming.

## 1. Introduction

The use of antibiotics growth promoters (AGPs) has been a common practice in intensive poultry production to improve animal growth performance, health, and sustainability of animal farming (1). However, AGPs threaten human health due to the risk of developing antimicrobial resistance and drug residue in poultry products (2). Consequently, AGPs have been prohibited in several countries, including the European Union, the United States, and China. However, AGPs are still frequently used in food animal production in developing countries (3). Among the used AGPs is zinc-bacitracin (ZnB), a mixture of high molecular weight polypeptides [bacitracin A, B, and C and various minor components (4, 5)]. However, the continuous use of ZnB induces suppression of natural immunity, dysbiosis, and antibiotic residues in animal products (6), highlighting the urgent need to find alternatives to AGPs, such as probiotics and prebiotics (7).

Probiotics are microorganisms, with beneficial effects as growth promoters and protectors against pathogen bacteria (8), by direct or indirect mechanisms (competitive exclusion), promoting host immunity and improving animal performance. Among probiotics is *Lactobacillus* spp. such as *L. acidophilus* (LA), which is promising in improving animal health and performance (8–10). Lactobacilli produce antibacterial proteins and bacteriocins (11), displaying a wide antibacterial spectrum against Gram-positive bacteria and improving chicken health (12). Muray et al. (13) suggested that probiotic supplementation containing *Lactobacillus* supports broilers’ growth similarly to diets supplemented with antibiotics and coccidiostats, improving the feed conversion rate. Yeasts also enhance feed quality and animal performance (14). The yeast mechanism of action includes two possible mechanisms: the first is related to supporting the growth of lactic acid bacteria, and the other is a competitive exclusion of pathogenic bacteria by yeast and its cell wall components (15). *S. cerevisiae* is also a source of protein, vitamin B-complex, enzymes such as cellulase and phytase, and trace minerals (16) and has a positive effect on mineral retention, bone mineralization, feed utilization, disease resistance, immune response, and growth performance of broilers (17).

It is also possible to use probiotics mixed with prebiotics (non-digestible feed ingredients able to stimulate the growth rate and/or the activity of some bacteria). This mixture, known as symbiotic, can enhance the activity and the survival of probiotics and stimulate bacteria living in the gastrointestinal tract, such as *Lactobacillus* and *Bifidobacteria* (18). Among prebiotics, mannan-oligosaccharides (MOS), a product derived from the outer cell wall of *S. cerevisiae*, are widely investigated, and their inclusion in poultry diets is of particular interest due to their positive effect on gut ecology and productive performance (19). In addition, the yeast cell wall has powerful antigenic stimulating properties, and it is well known that this property is a characteristic of the mannan chain (20).

Supplementation of poultry diets with MOS results in improved animal performance (21), partly due to its hypothesized nutrient-sparing effect and primarily due to its influence on nutrient utilization in the gut (22). According to Pascual et al. (23), supplementing broiler diets with *S. cerevisiae* cell wall improved animal health and performance. In addition, a significant improvement in antibody responses in broilers and layers due to MOS supplementation was also reported by other authors (24, 25). The positive impact of prebiotics over probiotic is still under scientific debate, and there are reports that probiotic alone is adequate (26). The present investigation aimed to evaluate the advantages of administering a probiotic supplement with *S. cerevisiae* or *L. acidophilus* and/or MOS prebiotic in terms of growth performance, digestibility, carcass traits, meat quality, and blood biochemistry for broiler chickens compared to those of zinc bacitracin.

## 2. Materials and methods

This work was approved by King Abdulaziz University, animal care and use committee office under institutional approval code ACUC-22-1-2.

### 2.1. Chickens and experimental design

A total of 150 one-day-old male Ross broiler chicks were wing banded and distributed based on similar initial body weight (44.6 g ± 1.6) among six groups. Each treatment was represented by 25 chicks/group with five replicates of five chicks of each. Each replicate was kept in battery brooders (35 × 25 × 30 cm). All groups were fed the same basal diet. The negative control group was fed the basal diet without any supplementation. Chicks kept in the ZnB group were supplemented with the basal diet supplemented with zinc bacitracin (ZnB group) 10% at 0.5 g/ kg diet (Pucheng Lifecome Biochemistry Co., Ltd. No.19, Nanpu Ecological Industrial Park, Pucheng, Fujian, P. R. China). Chicks kept in the “SC” group were fed the basal diet supplemented with a probiotic containing *S. cerevisiae* at 2 g/kg diet [China way Corporation Taiwan, 12^9^ colony-forming units (CFU) per gram]; However, chicks kept in the “LA” group were fed the basal diet supplemented with a probiotic containing *L. acidophilus* (5 × 10^11^ CFU/g) at 1 g/kg diet (Chinobio Trading Co., Ltd., Ningxia, China). Chicks kept in the “SC + MOS” group were fed the basal probiotic diets supplemented with *S. cerevisiae* at 2 g/kg diet and 1 g MOS /kg diet (Alltech Inc., Nicholasville, Kentucky, United States). However, chicks kept in the “LA + MOS group” were fed the basal diet supplemented with *L. acidophilus* at 1 g/kg and 1 g MOS /kg diet.

Chickens were fed a corn-soybean meal-based diet during starter (1–18 d), grower (19–28 d), and finisher (29–36 d) periods, formulated according to NRC (27) recommendations. The ingredients and chemical characteristics of the diets, determined according to AOAC (28), are shown in Table 1. Feed and water were provided *ad libitum*. Chicks were illuminated with a 23 light: 1 dark cycle and were vaccinated against Newcastle diseases (ND) using Hitcher B1 (at 7th day old) and Lasota (at 20 and 30 days of age) in the drinking water. At nine days old, all chicks were inoculated intramuscularly with inactivated Avian influenza subtype H5N2 (Nobilis^®^ Influenza H5N2, MSD Animal Health, Rahway, United States). Vaccination against the infectious bursal disease was done using live attenuated Gumboro vaccine (Nobilis^®^ Gumboro 228E, MSD Animal Health, Rahway, United States) at 14th and 24th -day-old.

**Table 1:** Ingredients and chemical composition of the experimental diets.

Ingredients, g/kg as feed	Starter diet	Grower diet	Finisher diet
Yellow corn	532.5	500.0	574.5
Soybean meal	384.3	373.0	315.0
Limestone	10.0	11.5	10.0
Dicalcium phosphate	20.0	17.0	16.0
Vit + Min Premix^a^	3.0	3.0	3.0
NaCl	3.0	3.0	3.0
DL-Methionine	3.9	3.0	2.5
L-Lysine (HCL)	3.3	1.4	1.3
Vegetable oils	40.0	71.5	64.0
Sand	0.0	16.6	10.7
Chemical-nutritional characteristics			
Dry matter^b^,%	89.85	89.78	89.78
ME kcal/kg^c^	3,022	3,153	3,205
Methionine^c^, %	0.72	0.62	0.55
Lysine^c^, %	1.43	1.24	1.09
Methionine+ Cystine^c^,%	1.07	0.96	0.86
Calcium^c^,%	0.97	0.96	0.86
Available phosphorus^c^,%	0.52	0.45	0.43
Crude protein^b^,%	21.87	20.89	18.83
Crude fibre^b^,%	3.79	3.71	3.41
Ash^b^,%	9.78	11.33	10.85
Ether Extract^b^,%	6.25	9.31	8.59
Nitrogen Free Extracts^b^,%	58.31	54.76	58.32

a Vit + Min mixture provides per kilogram of the diet: vitamin A (retinyl acetate) 24 mg, vitamin E (dl-α-tocopherol acetate) 20 mg, menadione 2.3 mg, Vitamin D3 (cholecalciferol) 0.05 mg, riboflavin 5.5 mg, calcium pantothenate 12 mg, nicotinic acid 50 mg, choline chloride 600 mg, vitamin B12 10 μg, vitamin B6 3 mg, thiamine 3 mg, folic acid 1 mg, d-biotin 0.50 mg. Trace mineral (milligrams per kilogram of diet): Mn 80 Zn 60, Fe 35, Cu 8, Se 0.60.

b Determined values.

c Calculated values.

### 2.2. Growth performance

During the experimental period, chickens of each group were individually weighed (g) (at 1th, 28th, and 36th days of age) in the morning, before offering feed; the body weight gain (at 1st, 28th, and 36th day of age), and the total weight from 1st to 36th days was calculated as the difference of the weight measured on the first and the last day of each period. At the same periods, feed intake was calculated as the difference between the feed (g) consumed on the first and last day of each period; thus, the feed conversion ratio (FCR) was calculated as feed intake/body weight gain (g/g) for each period. The European production efficiency index was calculated according to Huf et al. (29).

(1) $$\begin{array}{l} \mathrm{EPEF}=\mathrm{Body}\;\mathrm{weight}\;\left(\mathrm{kg}\right)\times \%\mathrm{viability}\times 100/\mathrm{feed}\\ \;-\mathrm{conversion}\;\mathrm{ratio}\times \mathrm{trial}\;\mathrm{duration}\;\mathrm{in}\;\mathrm{days}\text{.} \end{array}$$

### 2.3. Digestibility

The dry matter, organic matter, nitrogen-free extract (soluble carbohydrate), crude protein, crude fat, crude fiber, and apparent ash digestibility were measured at the end of the trial (36 d) using one chicken per replicate (5 chickens/group). The total faecal collection method was used. Chicks were fasted for 24 h, then fed on their corresponding experimental diets for 72 h, in which feed intake and voided excreta were accurately determined. The excreta samples were collected for each replicate, cleaned from feathers, and feed, weighed, and dried in a forced air oven at 70°C for 36 h. Samples were finally ground and placed in screw-top glass jars at 4°C until analyses. The procedure described by Jakobsen et al. (30) was used for separating faeces from urine nitrogen in excreta samples. Dry matter, nitrogen, fat, and crude fiber content of the excrement and feed were determined according to AOAC (28) and expressed on a dry matter basis. The apparent digestibility of nutrients was calculated by dividing the daily amount retained (g/d) by the amount intake (g/d). The daily amount of nutrient retained is equal to the amount of feed intake (% nutrient in feed × amount of feed consumed) minus that voided in the excreta (% nutrient in excreta, except for nitrogen which the fecal nitrogen was used ×amount of excreta voided).

### 2.4. Carcass quality

At 36 days of age, five chickens per treatment (1 per replicate) were weighed after fasting overnight, slaughtered, feather picked, and the total inedible parts (head, legs, and inedible viscera) were taken outside the carcasses, and then the remaining carcass was weighed. Abdominal fat was separated and weighed, including the fat in the abdominal cavity, and attached to the viscera. The internal organs were separated and weighed individually, including the liver, gizzard, heart, spleen, pancreas, and intestine. The intestinal length was measured (cm), and the carcass, abdominal fat, and internal organs weights were measured and expressed as percentages of live body weight.

Samples of meat, including 50% of breast +50% of thigh meat and liver samples (1 per replicate), were weighed and dried in an electric drying oven at 70° C for 24 h until constant weight. The dried flesh was finally ground using a suitable mixer to pass through a sieve (1 mm^2^) and then carefully mixed. The air-dried meat and liver samples were kept in a well-tight glass container for subsequent analysis. Dry matter, protein, ether extract, and ash were determined according to AOAC (28). The cholesterol content in meat and liver was determined using Sigma diagnostic cholesterol reagent procedure (No 352, Sigma Aldrich, St. Louis, MO, United States) (28).

The physical traits of meat samples were carried out using fresh samples (*n* = 5 per treatment). The water-holding capacity (WHC) and meat tenderness were measured according to Volvoinskaia and Kelman (31). The WHC was determined using 0.3 g minced meat tissues that were put under an ashless filter paper and pressed for 10 min. On the filter paper, two zones were formed. Their surface areas were measured by the planimeter. The WHC was calculated by subtracting the internal zone from the outer zone. The internal zone is due to the meat pressing only indicating tenderness.

The pH value was measured by a pH meter, as described by Aitken et al. (32). The pH was determined using 10.0 g of prepared samples from meat, and the drip was blended with 50 mL of distilled water for 10 min, and then the pH value was measured.

The color intensity of meat was determined according to the method of Husani et al. (33), as follows: 10 g of samples were shaken with 50 mL distilled water in a dark room for 10 min and then filtered, and the color intensity (absorbency) was measured photometrically at 543 mm.

### 2.5. Biochemical parameters

Five blood samples per group (one per replicate) were collected from wing veins in heparinized tubes at 36 d of age. The plasma was separated by centrifugation of blood at 1500 × *g* for 20 min and then stored at –20°C for further analyses. Biochemical constituents in plasma were determined using commercial kits (Plot No: 321, Sigma Diagnostics, POR Ramangamdi, Vadodara, India), as described by Al-Harthi et al. (34). The globulin concentration was estimated by subtracting albumin concentration from serum total protein.

### 2.6. Statistical analysis

Data were analyzed using one-way ANOVA of the statistical software SAS^®^ (35) according to the model: Yij = m + Di + eij, where Y is the value of the response variable, (m = the general mean), (i = dietary treatment), and (e = the error). The experimental unit was the pen/replicate for growth performance, while the single bird was the experimental unit for the other parameters such as nutrient digestibility, carcass and meat traits, and blood profiles. Before running the statistical analyses, the normality of data was tested using the Shapiro-Wilks test of normality (36). The mean difference at *p* ≤ 0.05 was tested using the Student–Newman–Keuls-test (36). The survival rate was assessed by using the chi-square test (36).

## 3. Results

### 3.1. Growth performance

During the entire trial, the mortality rate was very low (only three broilers died), and birds appeared in good satisfactory health condition. The performances of broilers during the entire trial are shown in Table 2. Different supplementations had no significant effect on broilers’ growth until day 28, but during 29–36 days, the SC group showed a significantly higher BWG (*p* ≤ 0.05) than the control and LA groups. Considering the entire period of the trial (1st–36th day), the body weight gain (BWG) of the SC group was higher (*p* ≤ 0.01) when compared with the control group. In the period from 1st–28th day of age, the feed intake of the SC + MOS group was lower (*p* ≤ 0.01) than the control and also the ZnB groups, and the LA group showed a significantly lower feed intake than that of the control (*p* ≤ 0.01). Between the 29th to 36th day of age, the following groups, SC, SC + MOS, and LA, had a significantly lower (*p* ≤ 0.01) feed intake compared to the control group. The FCR of the SC group was more favorable than the control (*p* ≤ 0.05) in the periods between the 29th–36th day of age and 1–36 d. However, no significant effects of all treatments were detected in comparison to the European production efficiency index, but the SC yielded a higher value than the LA and any combination of additives.

**Table 2:** Performance and European production efficiency index of broilers as affected by dietary treatment.

Item	Treatments						SEM	*p* value
	Control	SC	SC + MOS	LA	LA + MOS	ZnB		
**Body weight** (g)								
1 d	45.9	46.3	44.8	43.5	43.6	44.0	3.9	0.730
**Body weight gain** (g)								
1–28 d	1,018	1,028	1,021	1,047	1,015	1,007	25.5	0.860
29–36 d	571^b^	645^a^	615^ab^	579^b^	625^ab^	618^ab^	27.9	0.006
1–36 d	1,589^b^	1,673^a^	1,636^ab^	1,626^ab^	1,640^ab^	1,625^ab^	31.1	0.001
**Feed intake (g/chick)**								
1–28 d	1,749^a^	1,721^abc^	1,699^c^	1,703^bc^	1,718^abc^	1,734^ab^	7.3	0.001
29–36 d	1,204^a^	1,189^b^	1,181^b^	1,181^b^	1,197^ab^	1,194^ab^	3.9	0.003
1–36 d	2,953^a^	2,910^b^	2,880^b^	2,884^b^	2,915^b^	2,928^ab^	9.4	<0.001
**Feed conversion ratio**								
1–28 d	1.72	1.68	1.67	1.64	1.70	1.73	0.04	0.210
29–36 d	2.11^a^	1.84^b^	1.92^ab^	2.06^ab^	1.92^ab^	1.93^ab^	0.05	0.030
1–36 d	1.86^a^	1.74^b^	1.76^ab^	1.78^ab^	1.78^ab^	1.80^ab^	0.02	0.030
**Survival (%)**								
1–36 d	96	100	96	100	100	96	2.25	0.840
**European production efficiency index**								
1–36 d	228	267	248	254	256	241	8.9	0.470

^a, b,^ Means in the same row with various superscripts are significantly different (*p* < 0.05); *n* = 5 pens per group, each pen containing five birds; SC supplemented with *Saccharomyces cerevisiae*; LA supplemented with *Lactobacillus acidophilus*; MOS, mannan oligosaccharides; ZnB, Zinc bacitracin; SEM, standard error of the mean; EPEF = survivability % × average body weight (kg)/market age (day) × FCR (kg feed/kg gain) × 100.

### 3.2. Digestibility

Generally, the nutrient digestibility was unaffected by any of the above-mentioned treatments (Table 3). The data for carcass characteristics of broilers during the entire experimental period are shown in Table 4. The results indicated a statistically positive effect of dietary supplementations on the relative liver and spleen weight (*p* < 0.01). The liver % in the SC, LA, and ZnB groups was lower than that of the LA + MOS group. The spleen % in the SC group was lower than that of the control and ZnB groups, and that of the ZnB was higher than that of the LA group.

**Table 3:** Digestibility of nutrients (%) of broilers as affected by dietary treatments.

Items	Treatments						SEM	*p* value
	Control	SC	SC + MOS	LA	LA + MOS	ZnB		
Dry matter	80.5	80.4	80.1	81.0	80.3	80.9	0.73	0.95
Organic matter	82.3	81.6	81.4	82.3	82.1	82.7	0.65	0.89
Nitrogen–free extract	85.3	84.9	85.5	85.3	85.6	85.5	0.58	0.95
Crude protein	76.7	77.9	78.1	78.4	78.6	77.8	0.71	0.54
Ether extract	81.3	81.2	82.5	81.1	81.7	81.1	0.80	0.79
Crude fiber	35.6	36.9	37.1	37.1	36.1	36.6	0.59	0.38
Ash	32.7	32.0	31.2	30.5	33.1	33.1	0.18	0.83

SEM, standard error of means; *n* = 5 birds per group; SC supplemented with *Saccharomyces cerevisiae*; LA supplemented with *Lactobacillus acidophilus*, MOS, mannan oligosaccharides; ZnB, Zinc bacitracin; SEM, standard error of the mean.

**Table 4:** Carcass characteristics and internal body organs of 36 day-old broilers in % as affected by dietary treatments.

Items	Treatments						SEM	*p* value
	Control	SC	SC + MOS	LA	LA + MOS	ZnB		
Dressing, %	71.8	71.9	71.3	72.0	73.0	71.3	1.304	0.54
Offal,%	19.4	19.8	21.9	20.1	17.8	20.6	1.049	0.19
Breast, %	35.3	36.5	36.2	36.7	37.1	34.1	1.040	0.41
Thigh, %	29.8	30.8	32.0	31.1	31.9	33.6	1.085	0.24
Abdominal fat, %	0.31	0.69	0.24	0.44	0.36	0.59	0.22	0.69
Internal organ (%)								
Proventriculus,%	0.43	0.39	0.46	0.44	0.44	0.47	0.02	0.56
Gizzard,%	2.40	2.18	2.29	2.24	2.12	2.27	0.121	0.67
Liver, %	2.42^ab^	2.00^b^	2.55^ab^	2.18^b^	2.83^a^	2.30^b^	0.128	0.003
Heart,%	0.53	0.53	0.53	0.56	0.49	0.63	0.04	0.13
Spleen, %	0.13^ab^	0.08^c^	0.11^abc^	0.09^bc^	0.10^abc^	0.14^a^	0.01	0.006
Pancreas, %	0.11	0.11	0.25	0.23	0.26	0.22	0.02	0.44
Intestine length,%	8.70	9.37	8.31	9.37	9.06	9.86	0.60	0.43
Intestine weight,%	4.62	4. 73	5.51	4.73	4.50	5.54	0.482	0.69

^a, b^ Means in the same row with various superscripts are significantly different (*p* < 0.05); *n* = 5 birds per group; SEM, standard error of the means; SC supplemented with *Saccharomyces cerevisiae*, LA supplemented with *Lactobacillus acidophilus*, MOS, mannan oligosaccharides; ZnB, Zinc bacitracin.

### 3.3. Meat quality

Data for the chemical composition of liver and meat quality, including chemical composition and physical characteristics of 36 days old broilers, are shown in Table 5. The results showed no marked effect of different supplementations on most of the chemical composition analysis of the liver and the meat samples, except for liver cholesterol, cholesterol to lipid ratio, and meat cholesterol. All the dietary supplementations reduced cholesterol in the liver and meat compared to the control group (*p* < 0.01). In addition, the LA group had a cholesterol level in meat and liver lower than that of the SC group. All supplementations decreased the liver’s cholesterol-to-lipid ratio compared to the control group.

**Table 5:** Chemical composition as a percentage of the liver, chemical and physical characteristics of meat of 36 day-old broilers according to dietary treatments.

Items	Treatments						SEM	*p* value
	Control	SC	SC + MOS	LA	LA + MOS	ZnB		
Liver								
Dry matter, %	26.0	26.0	26.1	26.0	26.0	26.0	0.129	0.99
Protein, %	68.3	67.9	67.9	68.2	68.6	68.1	0.531	0.95
Lipid, %	23.9	24.0	24.3	23.8	23.6	23.9	0.424	0.97
Cholesterol, mg/g	2.98^a^	2.74^b^	2.62^c^	2.49^d^	2.54^cd^	2.62^c^	0.032	0.001
C/L ratio	12.8^a^	11.4^b^	10.81^b^	10.41^b^	10.60^b^	10.82^b^	0.265	0.001
Ash,%	6.38	6.44	6.30	6.27	6.46	6.31	0.166	0.89
Meat								
Dry matter, %	24.9	24.5	25.3	25.3	25.2	25.2	0.309	0.46
Protein, %	73.4	73.2	73.1	73.3	73.5	73.0	0.491	0.98
Lipid, %	19.0	19.5	19.1	19.5	18.9	19.1	0.473	0.91
Cholesterol, mg/g	88.2^a^	83.0 ^b^	81.2^bc^	78.8 ^c^	80.0^bc^	81.0^bc^	1.054	0.001
C/L ratio	0.465	0.428	0.429	0.403	0.424	0.426	0.014	0.099
Ash, %	5.88	5.71	5.73	5.50	5.78	5.94	0.179	0.61
Physical characteristics of meat								
pH	6.79	6.72	6.77	6.79	6.72	6.70	0.044	0.61
Color (Optical density)	0.163	0.166	0.175	0.164	0.155	0.166	0.008	0.74
Tenderness, 0.3 g/cm^2^	2.44	2.70	2.56	2.71	2.53	2.52	0.075	0.11
WHC, 0.3 g/cm^2^	4.75	4.92	4.78	5.08	4.76	4.81	0.087	0.086

^a, b^ Means in the same row with various superscripts are significantly different (*p* < 0.05); *n* = 5 birds per group; SC supplemented with *Saccharomyces cerevisiae*, LA supplemented with *Lactobacillus acidophilus*, MOS, mannan oligosaccharides, ZnB, Zinc bacitracin; SEM, standard error of the mean.

### 3.4. Biochemical parameters

Data for biochemical constituents of blood plasma of 36 days-old broiler chicks are shown in Table 6. The results revealed no significant effect of using different supplementations on most of the biochemical parameters of blood plasma except for plasma cholesterol and cholesterol-to-lipid ratio. In both cases, all the dietary treatments showed lower values than the control group (*p* ≤ 0.01).

**Table 6:** Blood plasma biochemical constituents of 36 days-old broilers according to dietary treatments.

Parameter	Treatments						SEM	*p* value
	Control	SC	SC + MOS	LA	LA + MOS	ZnB		
Total protein, g/dl	4.99	5.01	5.03	5.01	4.93	5.03	0.094	0.97
Albumin, g/dl	2.56	2.56	2.59	2.56	2.50	2.55	0.043	0.75
Globulin, g/dl	2.42	2.45	2.44	2.45	2.43	2.48	0.054	0.98
A/G ratio	1.062	1.047	1.065	1.027	1.044	1.031	0.013	0.22
Total lipid, mg/dl	700	705	704	703	702	694	0.095	0.97
Cholesterol, mg/dl	185^a^	170^b^	169^b^	166 ^b^	166 ^b^	168 ^b^	3.103	0.004
C/TL ratio	26.4^a^	24.1^b^	23.9^b^	23.6 ^b^	23.6^b^	24.2 ^b^	0.420	0.0009

^a, b^ Means in the same row with various superscripts significantly different (*p* < 0.05); *n* = 5 birds per group; SC supplemented with *Saccharomyces cerevisiae*; LA supplemented with *Lactobacillus acidophilus*; MOS, mannan oligosaccharides; ZnB, Zinc bacitracin; SEM, standard error of the mean.

## 4. Discussion

The observed good health condition of the birds has also been confirmed by metabolic profiles that, in all groups, fall within the physiological range of poultry species (35, 37). In the present trial, ZnB did not improve growth performance compared to the control group and produced a similar growth rate to the *S. cerevisiae* and *L. acidophilus* with or without MOS. However, Li et al. (38) observed that ZnB had a good growth performance compared with the probiotic-treated group. The divergent effects of ZnB may be attributed to the hygienic measures. Our previous studies indicated that ZnB, as a growth promoter, had no positive effects on performance when animals were kept under good sanitary conditions (37, 39).

Indeed, *S. cerevisiae* administered alone gave the best results in terms of performance which increased BWG and improved FCR compared to the control group. Although *S. cerevisiae* cannot attach to the intestinal epithelium, it remains active and flows through the gastrointestinal tract. It acts as a bioregulator via several mechanisms, including (i) detoxification of mycotoxins as well as other bacterial toxins and their receptors in the mucous membrane (40–42), (ii) improvement of gastrointestinal health by increasing the goblet cell densities and sizes (43, 44), (iii) providing some essential nutrients, such as vitamin B complex and several amino acids, and (iv) Improvement of nutrient digestion by providing cellulase and phytase. Akhavan-Salamat et al. (45) reported that yeast culture improved crude protein and mineral utilization in diets deficient in phosphors and thus enhanced P and Ca availability to the broilers, which could improve growth.

The lack of significant effect of *L. acidophilus* on the growth performance of broilers is in line with several studies (38, 39, 46), which reported that broilers’ growth, feed intake, and FCR were not affected by *Lactobacillus* spp. supplementations. Vicente et al. (47) reported that *Lactobacillus* spp. did not improve FCR and growth rate but significantly reduced the mortality of broilers. However, several studies reported significant positive effects of *L. acidophilus*. Jha et al. (48) found that chickens supplemented with different strains of *Lactobacillus* showed enhanced growth performance, gut histomorphology, and immune functions. In the same context, botanical probiotics containing *Lactobacillus* supplementations resulted in similar growth of broilers to that of the group supplemented with antibiotics and coccidiostat, but FCR was better (13). Moreover, Kalavathy et al. (49) found a significant increase in BWG and FCR of broilers supplemented with *Lactobacillus* cultures. These contradicting results might be attributed to the strain of lactobacilli and the hygienic measures.

Additionally, the lack of appreciated response to MOS supplemented over probiotic (*S. cerevisiae* or *L. acidophilus*) on the growth performance of broiler chicks for the entire rearing period is consistent with previous results. Yalcinkaya et al. (50) found that MOS supplementation at different concentrations (0.05%, 0.10%, and 0.15%) did not affect broiler growth rate, feed intake, and FCR during 1–42 days of age. Salehimanesh et al. (51) also reported no effects of a symbiotic based on *Lactobacillus* and MOS on feed intake, growth, FCR, carcass traits, intestinal morphology, and bacteria population of the ileum of broilers. However, other studies reported positive effects of synbiotics on the growth performance of broilers. Pelicano et al. (46) found that growth and FCR were significantly improved when MOS was added with probiotics such as *Bacillus subtilis* or *L. acidophilus* and *casei*, *Streptococci lactis* and *faecium*, *Bifidobacterium bifidum* and *Aspergillus oryzae*, but the improvements were only evident during 1st to 21 st days of age. Similarly, a mixture of *Lactobacillus* and *Aspergillus* fermentative products increased broilers’ BWG (52). This result was confirmed by Ghahri et al. (53), who observed an increase in feed intake and growth of broilers using a similar synbiotic in diets. The differences in the authors’ findings can be explained by considering the difference in the synbiotic type.

According to available publications, Shareef and Al-Dabbagh (10) and Attia et al. (8, 39) ZnB, *S. cerevisiae*, and *L. acidophilus* did not affect broilers’ carcasses and internal organs. However, some investigations found a significant reduction in abdominal fat using flavomycin (54) and small intestinal weight (55, 56). In addition, different supplementations induced various responses on the relative weight of the liver, which decreased due to *S. cerevisiae*, *L. acidophilus*, and ZnB supplementation. On the other hand, the spleen weight increased due to ZnB and decreased due to *S. cerevisiae* or *L. acidophilus* supplementation. These changes in the spleen as a secondary lymphoid organ can suggest lymphocyte production changes due to the above-mentioned supplementations. Lee et al. (57) reported that probiotics could inhibit enteric pathogens directly and indirectly via a competitive exclusion mechanism. *Lactobacilli* have also been reported to produce antibacterial proteins and bacteriocins (11), displaying a wide antibacterial spectrum against Gram-positive bacteria and enhancing chicken health (12). Thus, the positive effect of *S. cerevisiae* and *L. acidophilus* on the spleen could be explained based on the earlier evidence. On the other hand, Hock et al. (56) found that ZnB decreased the growth of clostridia, anaerobic cocci, enterococci, and coli-areogenic bacteria in the caecal contents of broilers. The lack of a significant effect of ZnB, *S. cerevisiae*, and *L. acidophilus* with or without MOS on carcass traits and meat quality agrees with the results of Attia (8) and Attia et al. (39).

In this study, it was found that plasma, meat, and liver cholesterol, as well as the cholesterol-to-lipid ratio of meat and liver, were significantly reduced in both the SC and LA groups when compared to the control group. The decrease in cholesterol and cholesterol to lipid ratio in plasma is in line with the decrease of both items in meat and liver (the metabolic side for lipids metabolism) in SC and LA groups, although *L. acidophilus* had a stronger effect. On the other hand, the lack of additive effect of MOS indicates that *S. cerevisiae* and *L. acidophilus* are adequate to control plasma, liver, and meat cholesterol. In the available pieces of literature, the cholesterol-lowering effect of probiotics has been quoted by several investigators (56–59). However, serum cholesterol was not affected when chickens were fed a probiotics-containing diet for three weeks of age, showing a time-dependent effect (60, 61). Similarly, probiotics supplementation (Lacto Sacc and Yea Sacc) significantly reduced total plasma cholesterol and lipids (62, 63). Supplementation of the diet with 1 and 2 g of Bio-Buds (dried SC fermentation product) decreased yolk and serum cholesterol and increased antibody production significantly (64). This reduction may be due to the ability of bacteria to assimilate or degrade the cholesterol to bile acids, followed by deconjugation to prevent re-synthesis. Additionally, Kalavathy et al. (49) found a significant decrease in serum triglycerides and lipids using *Lactobacillus* cultures. Yalcinkaya et al. (50) reported that cholesterol was significantly lower in the 0.05% MOS-fed group than in the other MOS groups (0.10% and 0.15%). Shareef and Al-Dabbagh (10) stated that *S. cerevisiae*, at 1%, 1.5%, and 2% decreased serum triglycerides and 2% for serum cholesterol.

The absence of significant changes in most of the plasma biochemical constituents due to ZnB, probiotics, or synbiotics are in partial agreement with the results of Attia et al. (6, 37) and Ashaverizadeh et al. (65); they reported that antibiotics and probiotics did not affect total protein, albumin, globulin, AST, ALT, triglycerides, cholesterol, HDL, LDL, and VLDL. However, Tollba et al. (66, 67) showed that probiotics (*Lactobacillus*, *Pediococuss*) significantly increased plasma protein, albumin, and globulin fractions in poultry reared under natural and heat-stress conditions. In addition, Abou El-Soud and El-Naggar (64) found that Natural yeast (SC) significantly increased the total serum protein and globulin levels. However, El-Ghamry and Fadel (59) found that *S. cerevisiae* and *Trichodermo reesei* did not affect the plasma total protein, albumin, and globulin. On the other hand, Abdel-Azeem et al. (62) found that probiotics supplementation increased total plasma protein, plasma albumin, and Ca; Shareef and Al-Dabbagh (10) stated that SC at 1%, 1.5%, and 2% increased total serum protein. The contradiction in response to probiotic supplementation among the above-mentioned investigations and that found herein could be elucidated based on the strain of bacteria, feed composition, and environmental and hygienic conditions (68, 69).

## 5. Conclusion

Broiler feed supplemented with dietary *S. cerevisiae* as probiotics performed similarly to zinc bacitracin-supplemented chicken feed and had significantly 5% higher growth, 39 points higher European production efficiency index, and 12 points reduced FCR compared to the non-supplemented control birds during the 36-d rearing period. Supplementing probiotics such as *L. acidophilus* improved the FCR by 8 points, but did not affect body weight (BWG) compared to the control group. However, both probiotics significantly reduced cholesterol levels in blood and meat, with the *L. acidophilus* reduction being more pronounced than *S. cerevisiae*. In general, probiotics had similar effects to zinc bacitracin on broiler performance, and MOS supplementation could not produce further improvements. It is also interesting to note that the control group showed similar values to the zinc bacitracin and the other additive groups. Our results show that using *S. cerevisiae* at 2 g/kg feed instead of antibiotics is possible to maintain healthy broiler farming and sustainability under intensive production.

## Data availability statement

The original contributions presented in the study are included in the article/supplementary material, further inquiries can be directed to the corresponding authors.

## Ethics statement

The animal study was approved by King Abdulaziz University, animal care and use committee office under institutional approval code ACUC-22-1-2. The study was conducted in accordance with the local legislation and institutional requirements.

## Author contributions

YA: Writing – original draft, Writing – review & editing. SB: Writing – original draft, Writing – review & editing. NA: Writing – original draft, Writing – review & editing. FB: Writing – original draft, Writing – review & editing. AA: Writing – original draft, Writing – review & editing. AS: Writing – original draft, Writing – review & editing. HH: Writing – original draft, Writing – review & editing.

## Funding

The author(s) declare financial support was received for the research, authorship, and/or publication of this article. This research work was funded by Institutional Fund Projects under grant no. (IFPIP: 451-155-1443).

## Conflict of interest

The authors declare that the research was conducted in the absence of any commercial or financial relationships that could be construed as a potential conflict of interest.

## Publisher’s note

All claims expressed in this article are solely those of the authors and do not necessarily represent those of their affiliated organizations, or those of the publisher, the editors and the reviewers. Any product that may be evaluated in this article, or claim that may be made by its manufacturer, is not guaranteed or endorsed by the publisher.
